# Feet deformities are correlated with impaired balance and postural stability in seniors over 75

**DOI:** 10.1371/journal.pone.0183227

**Published:** 2017-09-06

**Authors:** Ewa Puszczalowska-Lizis, Przemyslaw Bujas, Jaroslaw Omorczyk, Slawomir Jandzis, Marek Zak

**Affiliations:** 1 Institute of Physiotherapy, Faculty of Medicine, University of Rzeszow, Rzeszow, Poland; 2 Institute of Sport, Faculty of Physical Education and Sport, University School of Physical Education, Krakow, Poland; 3 Department of Physical Rehabilitation in Rheumatology and Geriatrics, Faculty of Physical Rehabilitation, University School of Physical Education, Krakow, Poland; University of Toronto, CANADA

## Abstract

**Objective:**

Understanding the factors and mechanisms that determine balance in seniors appears vital in terms of their self-reliance and overall safety. The study aimed to determine the relationship between the features of feet structure and the indicators of postural stability in the elderly.

**Methods:**

The study group comprised 80 seniors (41F, 39M; aged 75–85 years). CQ-ST podoscope and the CQ-Stab 2P two-platform posturograph were used as primary research tools. The data were analyzed based on Spearman’s rank correlation and forward stepwise regression.

**Results:**

Analysis of forward stepwise regression identified the left foot length in females and Clarke’s angle of the left foot in men as significant and independent predictors of postural up to 30% of the variance of dependent variables.

**Conclusions:**

Longer feet provide older women with better stability, whereas in men, the lowering of the longitudinal arch results in postural deterioration. In the elderly, the left lower limb shows greater activity in the stabilizing processes in the standing position than the right one. In gerontological rehabilitation special attention should be paid to the individually tailored, gender-specific treatment, with a view to enhancing overall safety and quality of seniors’ lives.

## Introduction

Motoric involution is a process that affects every human. It brings about an increasing incidence of falls and an attendant risk of injury in seniors. The structure and function of the foot are deemed crucial to the control of postural stability. From a biomechanical point of view, this part of the locomotor system is the actual area of contact between the body and the ground. Furthermore, the ankle joint is responsible for the most essential reactions involving corrective balance control, especially in the sagittal plane. The position of the centre of gravity (COG) relative to the boundaries of the plane of support may therefore be regarded as the determinant of stability in the standing posture [1]. The projection of the COG in the erect stance is located in a small, narrowly restricted area on the support plane–about 5 cm to the front of the lateral malleolus of the ankle joint, being subject to random shifts by about a dozen millimetres or so [2].

COG displacements and responses to the stimuli affecting the foot, also crucial for maintaining balance, are reflected in the oscillations of the centre of pressure (COP) [3]. In the seniors, the stability-impairing factors comprise multidirectional physiological involutionary processes [4–7], including structural and functional changes that occur in the nervous system, as well as in the active and passive parts of the motor system. Overall weakening of the bones, muscles and ligaments increases the incidence of musculoskeletal deformities, not least those affecting the feet. Consequently, this leads to functional limitations, pain, and falls-risk. Appreciating the mechanisms that determine balance will delay these adverse changes that affect postural stability and translates to patient’s overall self-reliance and safety.

The study aimed to determine the relationship between the features of feet structure and the indicators of postural stability in the elderly.

Research hypotheses:

1. Larger (longer and wider) feet provide better postural stability in older people.
2. Decrease of longitudinal and transverse foot arch adversely affects postural stability in older people.
3. The hallux valgus angle and the varus deformity of the fifth toe diminish postural stability in older people.

### Methods

We examined 80 senior community dwellers i.e. 41 female (mean age: 78.85±2.87 years) and 39 male (mean age:79.59±2.95 years) subjects. The subjects were randomly selected from among those who completed 75 years of age, were free living community dwellers at a purpose-built, housing estate for the seniors, as well as were found fully compliant with the inclusion criteria for the study. The following inclusion criteria were applied: age range of 75–85 years (*middle-old*), laterality determined on the basis of the *Waterloo Handedness and Footedness Questionnaire–Revised* [8], level of physical fitness that facilitates walking without any orthopaedic aids (canes, crutches, walkers), ability to assume a standing position on the podoscope and the stabilographic platform without any assistance, and a written informed consent to participate in the study.

Exclusion criteria:

- acute conditions (e.g. gastric pain due to intestinal obstruction, biliary colic, urinary tract infections, as well as musculoskeletal and muscular pain, etc.), which would make it unfeasible for a subject to adopt a free standing posture, while standing perfectly still on the platform during the testing protocol
- exacerbation of chronic conditions that might affect overall functional state during the study protocol (e.g. in the case of cardiovascular disorders, after a study subject has assumed a standing posture, orthostatic hypotension might set in)
- sensory disorders (e.g., when visual disturbances prevented focusing the eyes on a fixation point located 1 metre away; hearing impairments hampered and/or effectively precluded comprehension and execution of verbal commands)
- mental illnesses (e.g. dementia, disturbances of consciousness) or neurological conditions (e.g. cerebrovascular incidents, Parkinsonism)—making it unfeasible for a person to stand perfectly still during the testing procedure, while focusing his attention on a fixing point throughout
- regular administration of medications causing impairment of an individual sense of balance (e.g. antidepressants, antipsychotics; addiction to sedatives and/or sleep-inducing medications)
- alcohol addiction causing, inter alia, impairment of cognitive capacity, and/or interfering with an individual sense of balance.

Persons established (against their medical records and/or through interviews) to have sustained a fall within the 12 months preceding the actual date of the testing procedure have also been excluded from the study protocol.

The CQ-ST podoscope (manufactured by Electronic System) was made use of as the main research tool.

**Fig 1:**
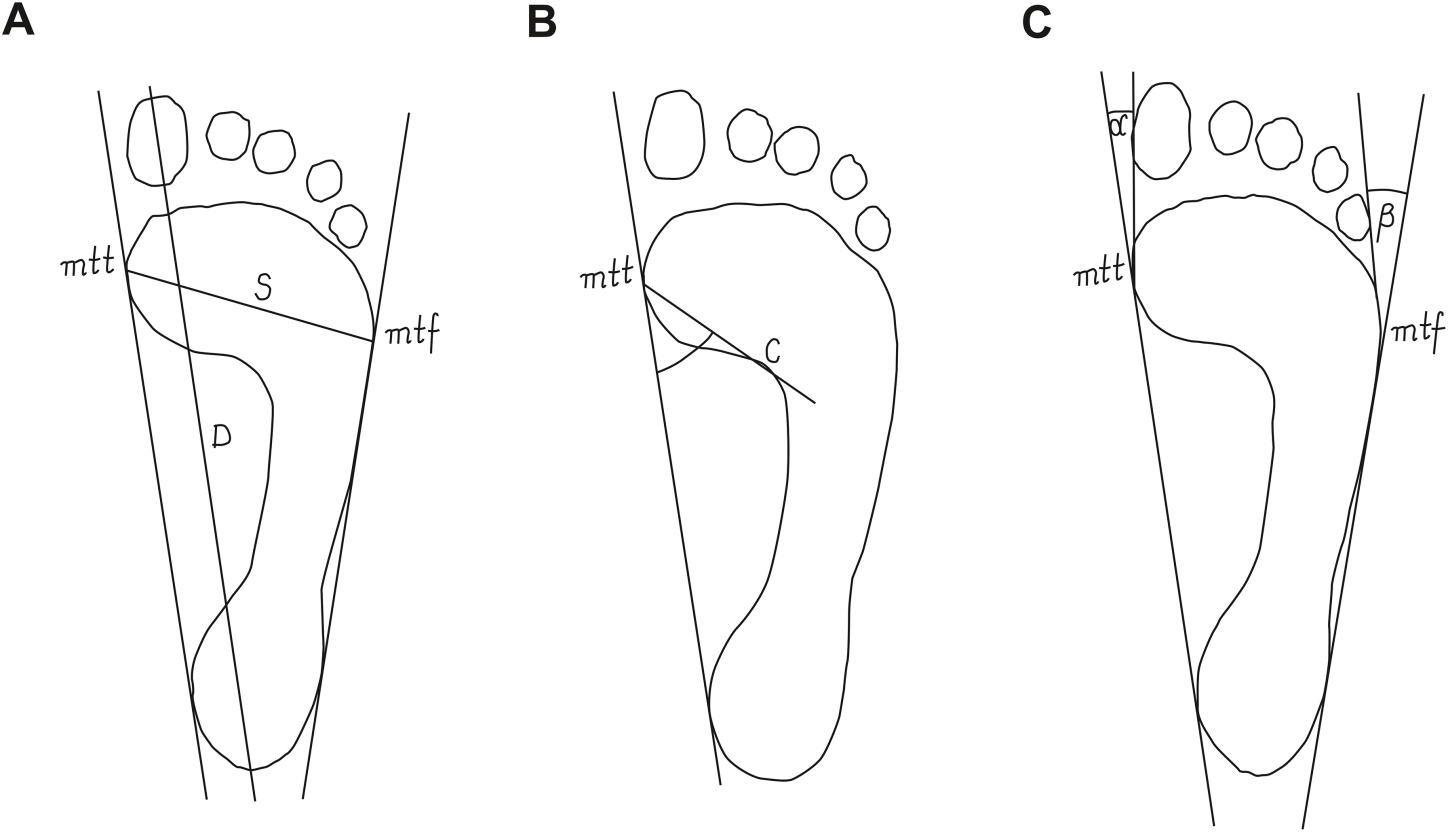
Procedure for determining the feet structure indices. (A) foot length, foot width and the Wejsflog index; (B) Clarke’s angle; (C) hallux valgus angle and the angle of the varus deformity of the fifth toe; mtt, metatarsale tibiale; mtf, metatarsale fibulare; D, foot length; S, foot width; C, point of the largest recess; α, hallux valgus angle, β, the angle of the varus deformity of the fifth toe.

The following parameters were measured:

- foot length–the line connecting the farthest points in the forefoot and the rearfoot, in cm (Fig 1A);
- foot width–the line connecting mtt (metatarsale tibiale) and mtf (metatarsale fibulare) points, in cm (Fig 1A);
- Clarke’s angle–defines the medial longitudinal arch (MLA)–it is located between the tangent to the medial edge of the foot and the line joining the point of the largest recess (point C) with the point of contact of the medial tangent to the forefoot (Fig 1B), in degrees;
- the Wejsflog (W) index (determines the transverse arch of the foot)–it is ratio of the length to the width of the foot (Fig 1A);
- hallux valgus angle (α)–(defines the position of the big toe)–it is located between the tangent line to the medial edge of the foot and the tangent to the pad of the big toe, derived from the mtt point (Fig 1C), in degrees;
- the angle of the varus deformity of the fifth toe (β)–(defines the position of the big toe)–it is located between the tangent line to the lateral edge of the foot and the tangent to the pad of the fifth toe, derived from the mtf point (Fig 1C), in degrees [9–14].

The procedures for calculating the feet structure indices are shown in Fig 1.

Stabilographic measurements were taken using the CQ-Stab 2P two-platform posturograph (CQ Electronic System). The device allowed simultaneous recording of the vertical centre of pressure position of the forces affecting each foot. Data from six sensors (three in each platform) were recorded. Sampling totalled 200 Hz per sensor. The platforms were levelled, their surfaces aligned in a single plane. The study entailed measuring postural stability in a relaxed stance, with the eyes wide open. The stance width and foot angle were natural, unforced. A fixation point was placed in front of the subject, at a distance of one meter. After getting onto the platform, the subject stood still, trying to maintain visual focus on the point of reference. The proper measurement was preceded by a 30-second "training" to stabilize the balance. Next, the test lasting 30 seconds was recorded. During the test, the investigator was positioned behind the subject.

The following indicators of stability were assessed:

- SPAP–statokinesiogram path length on the OY axis (sagittal plane), in mm;
- SPML–statokinesiogram path length on the OX axis (coronal plane), in mm;
- MAAP–mean COP displacement from the origin on the Y axis, in mm;
- MAML–mean COP displacement from the origin on the X axis, in mm;
- SA–sway area delimited by the COP point, in mm^2^;
- MF–mean frequency of COP displacement, in Hz;
- LWAP–number of COP displacements along the Y axis;
- LWML–number of COP displacements along the X axis.

The selection of stability indicators was based on the frequency of their use in the subject literature [15–18].

**Fig 2:**
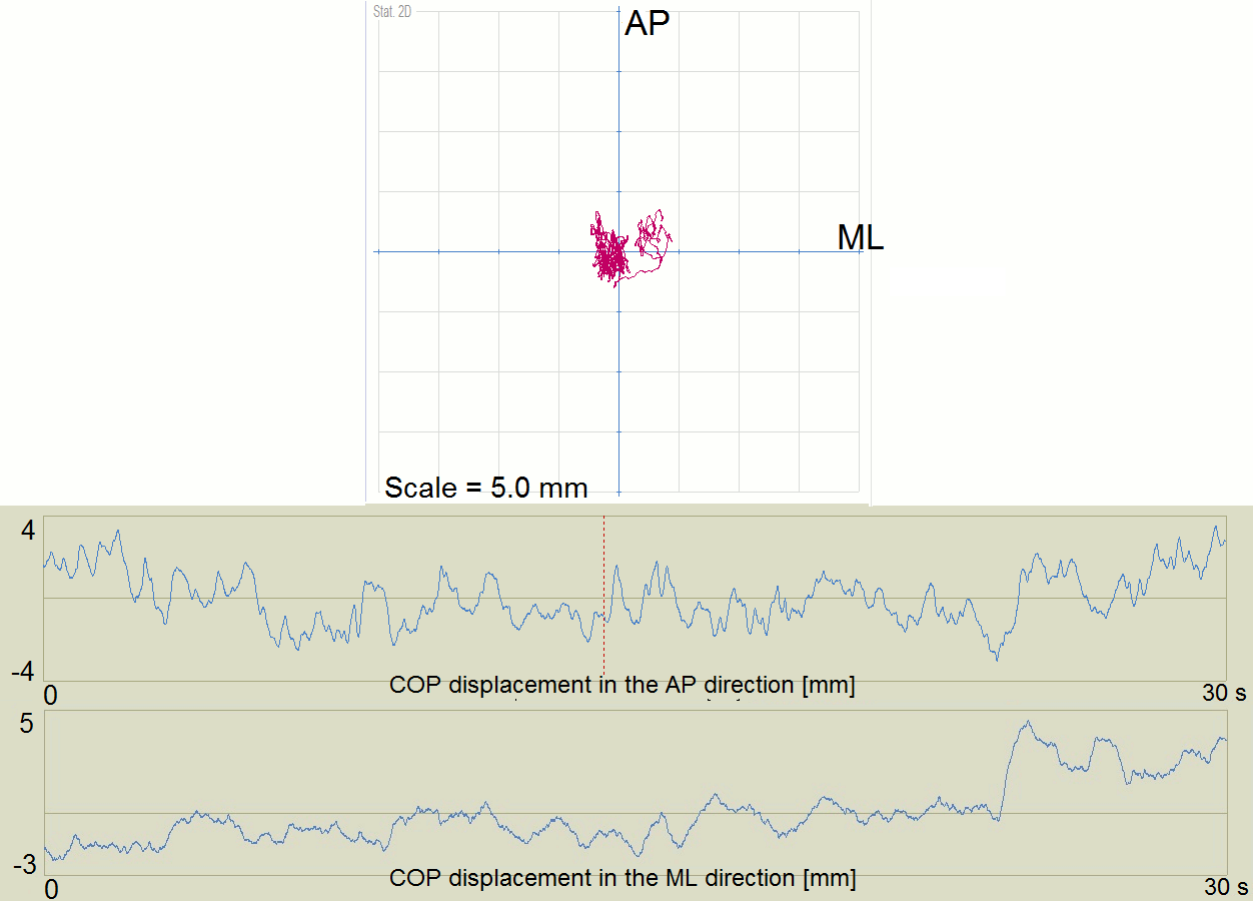
Example of the short path length for the COP. COP, Center of Foot Pressure; AP, anteroposterior direction; ML–mediolateral direction.

**Fig 3:**
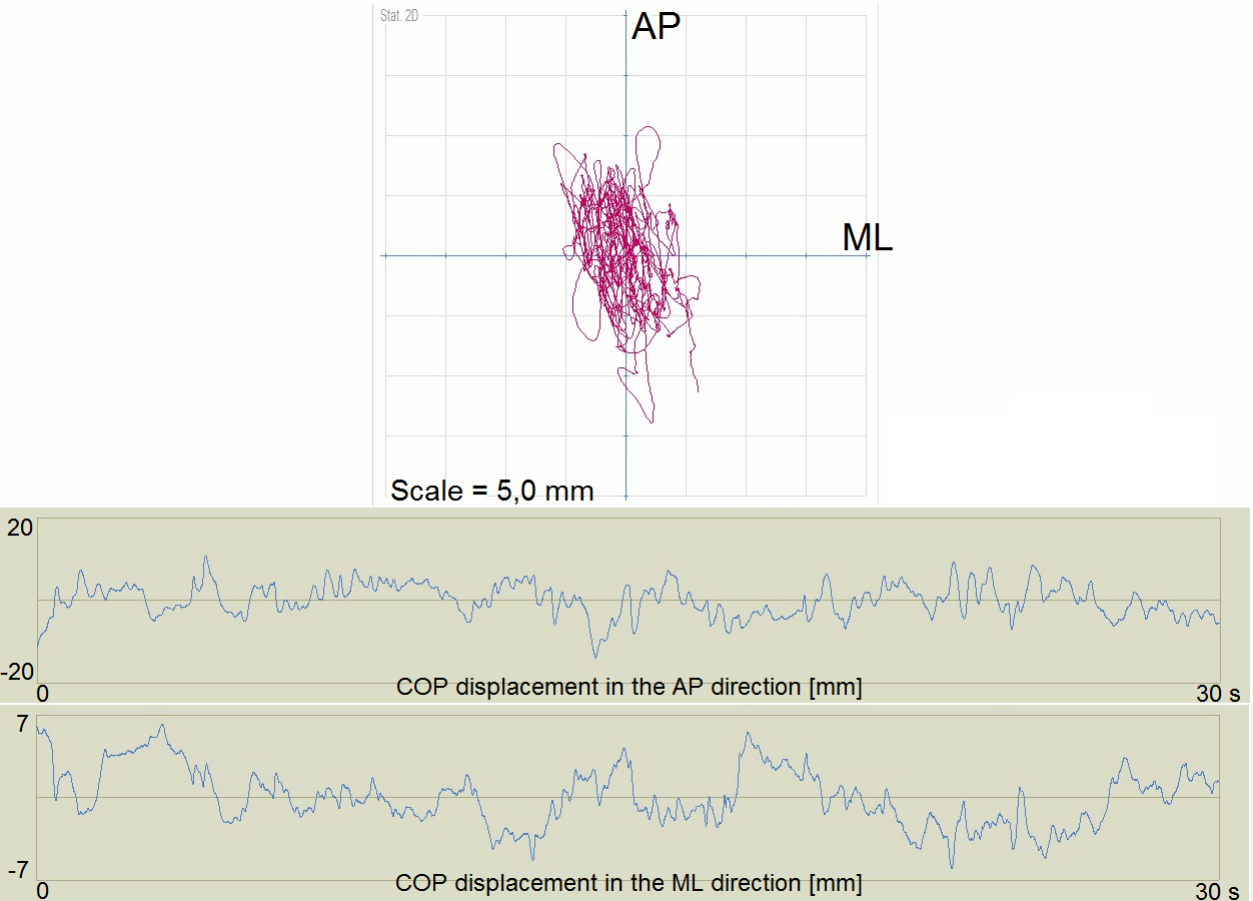
Example of the long path length for the COP. COP, Center of Foot Pressure; AP, anteroposterior direction; ML–mediolateral direction.

Examples of the short and long path length for the COP showed Figs 2 and 3.

Anthropometric measurements of the body mass and height were taken to calculate the Body Mass Index (BMI).

In order to ensure overall integrity of the research process, all tests were carried out in the morning, using the same measuring instruments operated by the Authors. The measurements were carried out in a gym, in the conditions facilitating elimination of any acoustic stimuli that might have interfered with postural reflexes during the study. Seniors were barefooted, dressed in sportswear.

The study was approved by a local academic Ethics Review Committee, The Bioethics Review Committee, University of Rzeszow (Approval Ref. No. 11/02/2013). All procedures were carried out in full compliance with the Declaration of Helsinki. All participants were furnished with detailed information on the aims and methods to be used throughout the study, and gave their written informed consent to participate.

### Statistical analysis

The consistency of the values with the normal distribution was verified by means of the Shapiro-Wilk test. In order to evaluate intersexes differences in average level of the basic somatic parameters we used the Student’s t test for independent samples. In order to evaluate the correlations between the structural characteristics of the feet and the stability indicators, Spearman’s rank correlation was applied. Forward stepwise regression analysis was used to predict the values of the balance indicators based on the parameters of the feet features. Only the variables found to be significantly associated with the balance indicators were entered into the regression analysis.

In the first step, only the independent variable found most strongly correlated with the dependent variable was selected and introduced into the model, thus identifying it with the most significant parameters. In the second step, another independent variable was selected whose values were strongly correlated with the remaining part of the first step, and the expanded model was characterized by the significance of all the parameters. Coefficient of determination (R^2^) was calculated, which represents the proportion of the total variation in the test score that is accounted for by the regression variables. The results were considered statistically significant, if the probability level of the test was lower than the predetermined significance level p<0.05. The Stat Soft STATISTICA application (version 10.0) was used to process the test results.

## Results

The basic somatic parameters of the study subjects are presented in Table 1.

**Table 1:** Somatic parameters of the study subjects.

Parameter	Women (n = 41)	Men (n = 39)	t	p-value ^a^
	mean±SD	mean±SD		
Body weight [kg]	69.12±16.06	80.47±12.96	-3,14	0,002*
Body height [cm]	153.88±9.25	168.62±5.97	-7.54	<0,001*
BMI	29.30±6.93	28.32±3.98	0.69	0.495

arithmetic mean; SD, Standard Deviation; t–value of the test statistic; BMI–Body Mass Index.

^a^ p-values were calculated using the Student’s t test for independent samples.

* p<0.05.

Table 2 lists the descriptive statistics pertaining to the structural parameters of the feet and the stability indicators observed in seniors under examination. These data indicate that the Clarke’s angle medians in both women and men have significantly lower values than those for adult population. In the case of the Wejsflog index, they were within the normal range, while the valgus angle of the toe and the varus angle of finger V were above the reference values.

**Table 2:** Descriptive statistics of structural features of the feet and the stability indicators in the study subjects.

Variable		Women (n = 41)		Men (n = 39)	
		Me	QR	Me	QR
**Foot structure parameters**					
Foot length [cm]	rf	22.50	1.60	24.60	1.20
	lf	22.50	1.50	24.80	1.30
Foot width [cm]	rf	9.10	1.10	9.40	0.90
	lf	9.00	0.90	9.40	1.00
Clarke’s angle [°] Reference values: 40–51^a^	rf	25.00	14.00	34.00	12.00
	lf	22.00	17.00	35.00	11.00
W index Reference values: 2–3^a^	rf	2.45	0.21	2.61	0.20
	lf	2.47	0.23	2.59	0.21
α angle [°] Reference values: 0–9^a^	rf	19.00	11.00	11.00	5.00
	lf	22.00	9.00	12.00	7.00
β angle [°] Reference values: 0–9^a^	rf	20.00	9.00	16.00	9.00
	lf	17.00	7.00	15.00	9.00
**Stability indicators**					
SPAP [mm]		240.00	138.00	249.00	165.00
SPML [mm]		151.00	79.00	161.00	57.00
MAAP [mm]		2.60	1.60	2.50	1.70
MAML [mm]		1.40	1.20	1.10	1.00
SA [mm^2^]		347.00	224.00	299.00	370.00
MF [Hz]		0.53	0.29	0.57	0.33
LWAP		19.00	18.00	23.00	17.00
LWML		15.00	10.00	20.00	11.00

Me, Median, QR, Quartile Range; rf, right foot; lf, left foot; SPAP, statokinesiogram path length on the OY axis; SPML, statokinesiogram path length on the OX axis; MAAP, mean COP displacement from the origin on the Y axis; MAML, mean COP displacement from the origin on the X axis; SA, sway area delimited by the COP point; MF, mean frequency of COP displacement, in Hz; LWAP, number of COP displacements along the Y axis; LWML, number of COP displacements along the X axis. R, Spearman’s rank correlation coefficient.

^a^ reference values cited by Lizis [11].

The data collected in Table 3 indicated that in women the length of each foot was in a statistically significant correlation with the mean displacement of the COP in the AP direction (R = 0.34 and p = 0.029 –right foot; R = 0.31 and p = 0.047 –left foot). Furthermore, there were negative correlations between the foot length and the number of COP displacements in the ML direction (R = -0.36 and p = 0.020 –right foot; R = -0.46 and p = 0.002 –left foot). The length of the left foot also negatively correlated with the mean COP displacement in the ML direction (r = 0.34, p = 0.028) and the average frequency of COP shifts (R = -0.42, p = 0.006).

**Table 3:** Correlations between structural features of the feet and the stability indicators in the female study subjects.

Variable		SPAP	SPML	MAAP	MAML	SA	MF	LWAP	LWML
Foot length (rf)	R p^a^	-0.02 0.956	-0.13 0.411	0.34 0.029*	0.25 0.113	0.21 0.198	-0.28 0.074	-0.11 0.465	-0.36 0.020*
Foot length (lf)	R p^a^	-0.15 0.357	-0.15 0.336	0.31 0.047*	0.34 0.028*	0.17 0.285	-0.42 0.006*	-0.25 0.113	-0.46 0.002*
Foot width (rf)	R p^a^	-0.12 0.454	-0.42 0.007*	0.14 0.400	0.09 0.556	-0.03 0.856	-0.19 0.229	-0.04 0.788	-0.14 0.384
Foot width (lf)	R p^a^	-0.10 0.519	-0.36 0.022*	0.26 0.098	0.14 0.396	0.04 0.789	-0.26 0.101	-0.16 0.327	-0.23 0.138
Clarke’s angle (rf)	R p^a^	0.14 0.382	0.27 0.092	-0.03 0.841	0.05 0.757	0.09 0.589	0.10 0.548	0.07 0.671	0.07 0.638
Clarke’s angle (lf)	R p^a^	0.02 0.917	0.17 0.282	-0.13 0.422	-0.09 0.586	-0.01 0.956	0.18 0.261	0.06 0.726	0.15 0.362
W index (rf)	R p^a^	0.07 0.665	0.34 0.028*	0.15 0.362	0.11 0.475	0.24 0.128	-0.02 0.908	-0.08 0.629	-0.15 0.354
W index (lf)	R p^a^	-0.12 0.468	0.16 0.302	-0.07 0.660	0.08 0.596	0.05 0.770	-0.07 0.643	-0.11 0.482	-0.17 0.283
α angle (rf)	R p^a^	0.04 0.787	-0.02 0.897	0.23 0.148	0.03 0.827	0.19 0.246	-0.13 0.870	-0.12 0.460	0.12 0.438
α angle (lf)	R p^a^	0.11 0.509	0.19 0.223	0.20 0.198	0.01 0.963	0.22 0.162	0.03 0.866	0.04 0.789	0.16 0.312
β angle (rf)	R p^a^	0.15 0.359	0.11 0.482	-0.09 0.551	0.22 0.158	0.06 0.695	0.06 0.726	0.33 0.032*	-0.13 0.421
β angle (lf)	R p^a^	0.26 0.100	0.22 0.170	-0.01 0.961	0.20 0.208	0.18 0.253	0.20 0.197	0.41 0.008*	-0.05 0.773

rf, right foot; lf, left foot; SPAP, statokinesiogram path length on the OY axis; SPML, statokinesiogram path length on the OX axis; MAAP, mean COP displacement from the origin on the Y axis; MAML, mean COP displacement from the origin on the X axis; SA, sway area delimited by the COP point; MF, mean frequency of COP displacement, in Hz; LWAP, number of COP displacements along the Y axis; LWML, number of COP displacements along the X axis; R, Spearman’s rank correlation coefficient.

^a^ p-values were calculated using the Spearman’s rank correlation.

* p<0.05.

Statistically significant negative correlations were found between the foot width and the statokinesiogram path length in the ML direction (R = -0.42 and p = 0.007 –right foot; R = -0.36 and p = 0.022 –left foot); the correlation between the Wejsflog index of the right foot and the statokinesiogram path length in the ML direction, on the other hand, was positive (R = 0.34, p = 0.028).

The angle of the varus deformity of the fifth toe of both feet correlated with the number of COP displacements in the AP direction (R = 0.33 and p = 0.032 –right foot; R = 0.41 and p = 0.008 –left foot).

The data in Table 4 indicated that with regard to men, the length of each foot was in a statistically significant correlation with the statokinesiogram path length in the AP direction (R = 0.38 and p = 0.016 –right foot; R = 0.46 and p = 0.003 –left foot) and the length of the right foot positively correlated with the size of the area encircled by COP (R = 0.32; p = 0.047). In the case of both feet, the Clarke’s angle correlated with the mean displacement of the COP in the AP direction (R = 0.34, p = 0.033 –right foot; R = 0.34 and p = 0.035 –left foot), mean displacement of the COP in the ML direction (R = 0.50, p = 0.001 –right foot; R = 0.39 and p = 0.015 –left foot) and with the mean frequency of COP displacement (R = -0.38, p = 0.018 –right foot; R = -0.41 and p = 0.010 –left foot). There was a statistically significant positive correlation between the Clarke’s angle of the right foot and the size of the area encircled by COP (R = 0.46; p = 0.003), and the Clarke’s angle negatively correlated with the number of COP displacements in the ML direction (R = -0.32 and p = 0.044). The hallux valgus angle (α) of the right and left foot positively correlated with the statokinesiogram path length in the ML direction (R = 0.36, p = 0.025 –right foot; R = 0.36 and p = 0.023 –left foot) and the size of the area encircled by COP (R = 0.33, p = 0.040 –right foot; R = 0.35 and p = 0.027 –left foot). The hallux valgus angle (α) of the right foot correlated with the mean COP displacement in AP direction (R = 0.35, p = 0.027).

**Table 4:** Correlations between structural features of the feet and the stability indicators in the male study subjects.

Variable		SPAP	SPML	MAAP	MAML	SA	MF	LWAP	LWML
Foot length (rf)	R p^a^	0.38 0.016*	0.19 0.235	0.16 0.343	0.26 0.112	0.32 0.047*	0.10 0.555	0.19 0.234	-0.16 0.323
Foot length (lf)	R p^a^	0.46 0.003*	0.23 0.160	0.26 0.109	0.33 0.043	0.39 0.015	0.05 0.753	0.16 0.321	-0.14 0.402
Foot width (rf)	R p^a^	0.14 0.385	0.06 0.734	0.15 0.374	0.27 0.096	0.29 0.076	0.01 0.942	0.07 0.654	-0.11 0.487
Foot width (lf)	R p^a^	-0.01 0.949	0.07 0.967	0.11 0.501	0.20 0.223	0.16 0.319	-0.05 0.737	-0.08 0.626	-0.11 0.492
Clarke’s angle (rf)	R p^a^	-0.16 0.925	0.21 0.208	0.34 0.033*	0.50 0.001*	0.46 0.003*	-0.38 0.018*	-0.26 0.105	-0.32 0.044*
Clarke’s angle (lf)	R p^a^	-0.07 0.664	0.03 0.867	0.34 0.015*	0.39 0.015*	0.31 0.053	-0.41 0.010*	-0.21 0.207	-0.20 0.220
W index (rf)	R p^a^	0.16 0.324	0.17 0.310	0.11 0.493	0.04 0.788	0.11 0.514	0.03 0.874	-0.01 0.935	-0.13 0.427
W index (lf)	R p^a^	0.37 0.020	0.29 0.072	0.17 0.293	0.13 0.431	0.26 0.114	0.13 0.437	0.17 0.306	0.02 0.888
α angle (rf)	R p^a^	0.26 0.113	0.36 0.025*	0.35 0.027*	0.13 0.434	0.33 0.040*	-0.03 0.850	0.06 0.704	0.29 0.072
α angle (lf)	R p^a^	0.12 0.481	0.36 0.023*	0.22 0.177	0.18 0.276	0.35 0.027*	-0.06 0.711	-0.09 0.604	0.28 0.085
β angle (rf)	R p^a^	0.00 0.983	0.11 0.493	0.04 0.825	0.14 0.378	0.11 0.490	-0.06 0.717	0.09 0.579	-0.04 0.788
β angle (lf)	R p^a^	-0.28 0.084	-0.17 0.292	-0.03 0.835	0.02 0.882	-0.11 0.519	-0.10 0.548	-0.17 0.305	-0.18 0.274

rf, right foot; lf, left foot; SPAP, statokinesiogram path length on the OY axis; SPML, statokinesiogram path length on the OX axis; MAAP, mean COP displacement from the origin on the Y axis; MAML, mean COP displacement from the origin on the X axis; SA, sway area delimited by the COP point; MF, mean frequency of COP displacement, in Hz; LWAP, number of COP displacements along the Y axis; LWML, number of COP displacements along the X axis.

^a^ p-values were calculated using the Spearman’s rank correlation.

* p<0.05.

Table 5 presents statistically significant regression models. These data indicate that in the case of women, the variation of the statokinesiogram path length in the ML direction in 12% was accounted for by the variation of the left foot width (R^2^ = 0.12). In turn, the left foot length was the factor accounting for the 33% of the variance of the dependent variable termed the mean frequency of COP displacement (R^2^ = 0.33), as well as the 32% variance of the variable: the number of COP displacements in the ML direction (R^2^ = 0.33) and 32% variance of the variable: the number of COP displacements in the ML direction (R^2^ = 0.32).

**Table 5:** Statistically significant models of forward stepwise regression.

Dependent variable	Predictor variable	Value of the regression model	R^2^
Female			
SPML	Foot width (lf)	F (1.39) = 5.30; p = 0.027*	0.12
MF	Foot length (lf)	F (1.39) = 19.10; p<0.001*	0.33
LWML	Foot length (lf)	F (1.39) = 18.22; p<0.001*	0.32
Male			
SPAP	Foot length (lf)	F (1.37) = 4.58; p = 0.039*	0.11
SPML	α angle (lf)	F (1,37) = 4.61; p = 0.038*	0.11
MAML	Clarke’s angle (rf)	F (1.37) = 8.25; p = 0.007*	0.18
SA	Clarke’s angle (rf)	F (1.37) = 8.35; p = 0.006*	0.18
MF	Clarke’s angle (lf)	F (1.37) = 16.52; p<0.001*	0.31

rf, right foot; lf, left foot; SPAP, statokinesiogram path length on the OY axis; SPML, statokinesiogram path length on the OX axis; MAAP, mean COP displacement from the origin on the Y axis; MAML, mean COP displacement from the origin on the X axis; SA, sway area delimited by the COP point; MF, mean frequency of COP displacement, in Hz; LWAP, number of COP displacements along the Y axis; LWML, number of COP displacements along the X axis; R^2^, coefficient of determination; F, the Fisher-Snedecor test.

* p<0.05.

In men, the length of the left foot was a predictor variable for the statokinesiogram path length in the AP direction, and the hallux valgus angle (α) of the left foot was the explanatory variable for the statokinesiogram path length in the ML direction. In both cases, the regression models accounted for 11% of the variance of the dependent variable (R^2^ = 0.11). The Clarke's angle of the right foot was the predictive variable for the COP mean displacement in the ML direction, and for the size of the area encircled by the COP. In each model, the predictor variable accounted for 18% of the variance of the dependent variable (R^2^ = 0.18). In turn, the Clarke's angle of the left foot was the predictor variable for the mean frequency of COP displacement, accounting for 31% of the variance of the dependent variable (R^2^ = 0.31).

## Discussion

The findings of this study demonstrate there is a significant association between foot characteristics and performance in balance in older people. The final considerations were based on the results of regression analysis, which allowed to extract the variables explaining the analyzed stability indices. In women, the width of the left foot was found to be a predictor variable for the statokinesiogram path length in the mediolateral direction. Furthermore, the left foot length was a predictor of the mean frequency of COP displacement and the mean displacement of the COP in the ML direction. Wider feet were associated with shorter COP path towards ML, whereas longer feet were associated with the lower frequency of corrective response and a smaller number of COP displacements in the ML direction. The relations at issue pertained to the left foot, which seems indicative of a predominant role of this foot in maintaining balance. It was also found that in men the length of the left foot was an independent predictor for the statokinesiogram path length toward AP. It follows that longer feet were associated with a longer COP path in the AP direction, and it also indicates a stronger impact of the ankle strategy on the stability control in men. The rationale for such correlations may be found in the report by Blaszczyk et al. [19], which indicates that postural reactions become slower with age. The consequences consist in the deterioration of stability indicators resulting from the generation of larger values of ground reaction forces in the case of delayed reactions in response to postural sways. Likewise, Simoneau et al. [20] indicate that the age-related changes comprise the increases in the amplitude and frequency of postural sway in the AP and ML dimensions. Older adults have a larger magnitude, speed, area, and variability of sway than the younger ones. Furthermore, the frequency range and mean power frequency of sway is higher in the older adults, as compared to the younger ones [21]. These changes suggest that older adults have difficulty controlling their sway, as the COM is allowed to drift further toward the limits of stability, thus requiring larger stabilizing moments to be generated in order to maintain an upright stance. The results yielded by our study do not corroborate the observations of Chiari et al. [17] who, in the study of young adults, considered the foot length and width to be the variables of no impact on the values of stability indices.

In men, the main predictor variable was the Clarke's angle. As to the left lower limb, which has a stabilizing function, the flattening of the foot resulted in a higher frequency of corrective shifts in foot pressure. These data are also corroborated by other authors. Wright at al. [22] observed the effect of flatfootedness on the increased speed of COP displacements in the subjects aged 26–44. Based on the research conducted with the aid of the sway meter, Spink et al. [23] demonstrated that a foot deformity was associated with an increased area of centre-of-gravity sways in persons aged 65–93. According to the above referenced report, foot posture (measured using the FPI) is an independent predictor of postural sway on foam. A more pronated (flatter) foot is thus believed to correspond to poorer performance. Menz et al. [18] determined that FPI and AI were significantly associated with balance, whereas hallux valgus was not an independent predictor in terms of balance in seniors. In the case of the right lower limb, which has a manipulative function and demonstrates readiness for defensive reactions, the Clarke’s angle accounted for the shaping of the variables: mean of COP displacement in the ML direction, and sway area delimited by the COP. The lowering of the longitudinal arch was associated with a decrease in mean COP displacements towards ML, as well as a decrease in the field of the ellipse plotted by the COP.

The influence of toe positions on COP trajectory seems to be well worth considering. Toes I and V, which are responsible for the equal distribution of the pressure forces controlled by the trochlea tali, are particularly noteworthy. According to numerous authors, biomechanical abnormalities that affect the forefoot as a result of degenerative changes generate strong pain, cause the formation of corns and calluses, and change the static foot posture. Koller et al. [24] conducted studies of people with hallux valgus aged 20–79, and found negative correlations between the hallux valgus angle and the peak pressure in the big toe, and positive correlations between the force time integral, contact area, maximum force and the peak pressure on the head of the fifth metatarsal. Martínez-Nova et al. [25] found that the progression of the hallux valgus generates an increase in pressure on the ground beneath its plantar surface. On the other hand, Ferrari et al. [26] did not encounter any correlation between the inclination angle of the big toe and the pressure on the first metatarsal head. According to Wen et al. [27], hallux valgus causes the load to shift away from the first metatarsal head towards the heads of the second and third metatarsal bones. Okuda et al. [14] found statistically significant correlations between the hallux valgus angle and painfulness of the feet which caused greater discomfort as the deformity progressed, consequently translating into overall postural instability. Menz and Lord [28] recognize foot pain as one of the factors that have an adverse impact on balance and functional abilities in the aged people. Our findings indicate that forefoot deformation in male subjects may be related to postural stabilization. The α angle of the left foot was a predictive factor for statokinesiogram path length in the ML direction. Progressing deformity of halux valgus was accompanied by a longer path in the ML direction. It is suggested, that the lateral tilts may be relied on as a sort of defensive strategy, adopted with a view to compensating for the accumulation of symptoms associated with degenerative changes and the distortion of the forefoot. Slight elevation of the toes above the ground is jointly instrumental for relieving the pressure on the head of the I metatarsal bone.

It may well be ventured to say that feet deformities decrease overall stability. The foot types selected through the regression analysis account for the variability of the stability indexes under study within the 11%–33% range. The main limitations of our study consisted in the fact that overall functioning of the balance control system is dependent upon the feedback from different functional and anatomical systems, which makes it rather hard to select the specific variables that may impact its work independently; this being particularly difficult in the case of the elderly affected by the involutionary changes. Despite these limitations, the results bring potentially significant implications for the elderly. In structuring the final conclusion we considered those anatomical features of the feet in respect of which the determinant coefficients showed conformity with the model in excess of 30%. It may therefore be assumed that the left foot length in women, and the Clarke’s angle of the left foot in men are the strongest predictors for the mean frequency of COP displacement. In women, the left foot length was also the predictor for the number of COP displacements in the ML direction. It follows that that the left lower limb is much more instrumental in the standing position stabilizing activities than the right one. This does not imply, however, that this type of structural adaptive mechanism should prove effective in every circumstances. In dynamic conditions, when the shape of the foot is subject to constant adjustments, making use of some additional mechanisms that support and control balance, proves essential. Consequently, kinesiogerontoprofilactic therapy should primarily take into account the actions aimed at the introduction of optimal preventive measures against the age-related loss of feet effectiveness. Bearing in mind the age-related constraints, optimal functionality would be ensured by suitably selected footwear, specifically designed to conform with the age-related changes in the foot shape. This type of footwear should be capable of lifting the medial margin of the foot, prevent deformations, corns, and pain in the forefoot. Passive correction of foot setting should be supported by the exercises specifically aimed at increasing the capacity of the corresponding muscles and elasticity of periosteal structures, thus enhancing overall mobility of joints and proprioception. General fitness and sensorimotor exercises are also essential, including the low-stability training (e.g. exercises on one leg, or on an unstable surface), Tai-chi, and outdoor leisure activities at large.

## Conclusion

Longer feet provide better stability in older women, as they coexist with the lower frequency of corrective response, and fewer COP displacements towards ML. In men, the lowering of the longitudinal arch in the left foot adversely affects overall stability, as it coincides with an increase in the frequency of the corrective responses. In the elderly, the left lower limb is much more instrumental in the standing position stabilizing activities than the right one. In gerontological rehabilitation, special attention should be paid to the individually tailored, gender-specific treatment, with a view to enhancing overall safety and quality of seniors’ life.

## Supporting information

S1 Dataset (XLS)
